# Crystal structure of 4-methyl­benzyl *N*′-[(thio­phen-2-yl)methyl­idene]hydrazinecarbodi­thio­ate

**DOI:** 10.1107/S205698901501107X

**Published:** 2015-06-13

**Authors:** Syahirah binti Ramli, Thahira Begum S. A. Ravoof, Mohamed Ibrahim Mohamed Tahir, Edward R. T. Tiekink

**Affiliations:** a Department of Chemistry, Universiti Putra Malaysia, 43400 Serdang, Malaysia; b Department of Chemistry, University of Malaya, 50603 Kuala Lumpur, Malaysia

**Keywords:** crystal structure, hydrogen bonding, di­thio­carbazate, C—H⋯π inter­actions

## Abstract

In the title compound, C_15_H_16_N_2_S_3_ {systematic name: [({[(4-methyl­phen­yl)meth­yl]sulfan­yl}methane­thio­yl)amino][1-(thio­phen-2-yl)ethyl­idene]amine}, the central CN_2_S_2_ residue is almost planar (r.m.s. deviation = 0.0061 Å) and forms dihedral angles of 7.39 (10) and 64.91 (5)° with the thienyl and *p*-tolyl rings, respectively; the dihedral angle between these rings is 57.52 (6)°. The non-thione S atoms are *syn*, and with respect to the thione S atom, the benzyl group is *anti*. In the crystal, centrosymmetrically related mol­ecules self-associate *via* eight-membered {⋯HNCS}_2_ synthons. The dimeric aggregates stack along the *a* axis and are are consolidated into a three-dimensional architecture *via* methyl-C—H⋯π(benzene) and benzene-C—H⋯π(thien­yl) inter­actions.

## Related literature   

For the structure of the parent compound, in which the benzyl residue is *syn* to the thione S atom, see: Chan *et al.* (2003 ▸). For the synthesis, see: Tarafder *et al.* (2002 ▸).
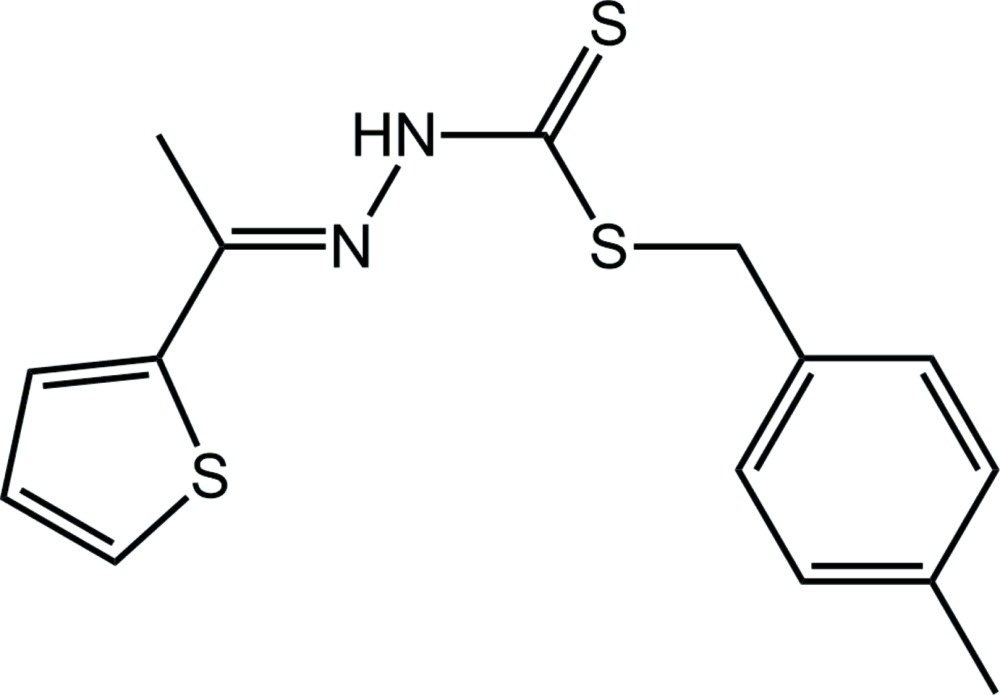


## Experimental   

### Crystal data  

C_15_H_16_N_2_S_3_
*M* *_r_* = 320.48
Monoclinic, 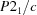

*a* = 5.6956 (4) Å
*b* = 14.3424 (9) Å
*c* = 18.9255 (11) Å
β = 90.263 (5)°
*V* = 1545.98 (17) Å^3^
*Z* = 4
Cu *K*α radiation
μ = 4.30 mm^−1^
*T* = 150 K
0.15 × 0.10 × 0.06 mm

### Data collection  

Oxford Diffraction Xcaliber Eos Gemini diffractometer
Absorption correction: multi-scan (*CrysAlis PRO*; Agilent, 2011 ▸) *T*_min_ = 0.774, *T*_max_ = 1.000
8463 measured reflections
2830 independent reflections
2506 reflections with *I* > 2σ(*I*)
*R*_int_ = 0.023

### Refinement  

*R*[*F* ^2^ > 2σ(*F* ^2^)] = 0.039
*wR*(*F* ^2^) = 0.109
*S* = 1.06
2830 reflections
186 parameters
1 restraint
H atoms treated by a mixture of independent and constrained refinement
Δρ_max_ = 0.49 e Å^−3^
Δρ_min_ = −0.33 e Å^−3^

### 

Data collection: *CrysAlis PRO* (Agilent, 2011 ▸); cell refinement: *CrysAlis PRO*; data reduction: *CrysAlis PRO*; program(s) used to solve structure: *SHELXS97* (Sheldrick, 2015 ▸); program(s) used to refine structure: *SHELXL2014* (Sheldrick, 2015 ▸); molecular graphics: *ORTEP-3 for Windows* (Farrugia, 2012 ▸) and *DIAMOND* (Brandenburg, 2006 ▸); software used to prepare material for publication: *publCIF* (Westrip, 2010 ▸).

## Supplementary Material

Crystal structure: contains datablock(s) 1, I. DOI: 10.1107/S205698901501107X/hb7439sup1.cif

Structure factors: contains datablock(s) I. DOI: 10.1107/S205698901501107X/hb7439Isup2.hkl

**Fig. 1.**
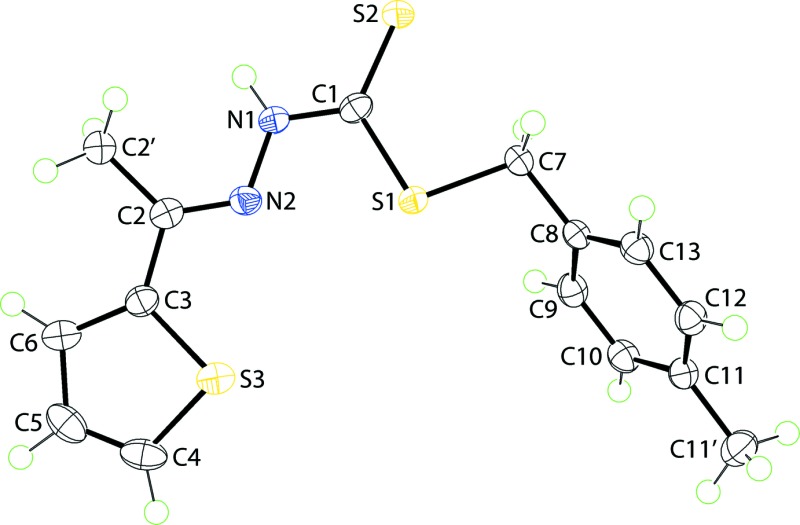
The molecular structure of the title compound showing displacement ellipsoids at the 70% probability level.

. DOI: 10.1107/S205698901501107X/hb7439fig1.tif
The mol­ecular structure of the title compound showing displacement ellipsoids at the 70% probability level.

**Fig. 2.**
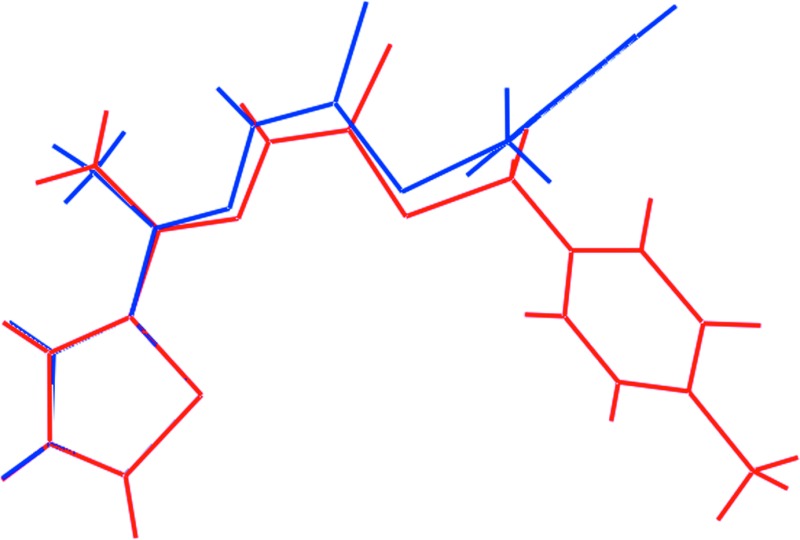
Overlay diagram of the title compound (red image) with the parent compound (blue). The molecules have been overlapped so that the thienyl residues are coincident.

**Fig. 3.**
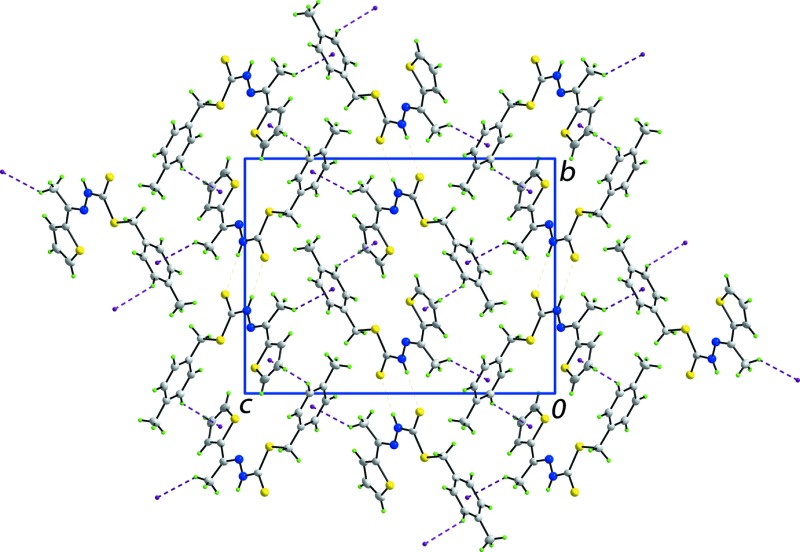
A view of the unit-cell contents in projection down the a axis. The N—H···S (orange) and C—H···π (purple) interactions are shown as dashed lines.

. DOI: 10.1107/S205698901501107X/hb7439fig2.tif
Overlay diagram of the title compound (red image) with the parent compound (blue). The mol­ecules have been overlapped so that the thienyl residues are coincident.

a . DOI: 10.1107/S205698901501107X/hb7439fig3.tif
A view of the unit-cell contents in projection down the *a* axis. The N—H⋯S (orange) and C—H⋯π (purple) inter­actions are shown as dashed lines.

CCDC reference: 1405284

Additional supporting information:  crystallographic information; 3D view; checkCIF report

## Figures and Tables

**Table 1:** Hydrogen-bond geometry (, ) *Cg*1 and *Cg*2 are the centroids of the S3,C3C6 and C8C13 rings, respectively.

*D*H*A*	*D*H	H*A*	*D* *A*	*D*H*A*
N1H1*N*S2^i^	0.87(2)	2.57(2)	3.4433(18)	176(3)
C2H22*Cg*2^ii^	0.98	2.85	3.616(3)	138
C12H12*Cg*1^iii^	0.95	2.89	3.560(2)	130

Symmetry codes: (i) 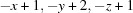; (ii) 
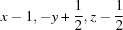
; (iii) 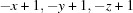.

## supplementary crystallographic information

### S1. Experimental

The title compound was prepared as per a reported procedure (Tarafder *et al.*, 2002). The light-yellow precipitate formed was filtered off and recrystallized from its acetonitrile solution as yellow prisms. Yield 56%; *M*.pt: 175–177 °C. Anal. Calcd for C_15_H_16_N_2_S_3_: C, 56.21; H, 5.03; N, 8.74. Found: C, 55.97; H, 4.96; N, 8.10. IR (cm^-1^, FT—IR): 3143 w, 1511 m, 1060 m, 924 s. ^1^H-NMR: (DMSO-d_6_, p.p.m.) δ: 12.42 (s, 1H, NH), 7.24–7.55 (multiplet, 4H, Ar–H), 7.03–7.10 (multiplet, 3H, thiophene-H), 4.37 (s, 2H, –SCH_2_), 2.24, 2.36 (s, 6H, –CH_3_), 13 C-NMR:(DMSO-d_6_, p.p.m.) δ: 197.98 (C=S), 159.15 (C=N), 129.32–142.86 (Ar–C), 128.39–129.90 (thiophene-C), 38.23 (SCH_2_), 15.58, 21.24 (CH_3_).

### S2. Refinement

Carbon-bound H-atoms were placed in calculated positions (C—H = 0.95 to 0.99 Å) and were included in the refinement in the riding model approximation with *U*_iso_(H) = 1.2–1.5*U*_eq_(C). The N—H atom was refined with N—H = 0.88±0.01 Å, and with *U*_iso_(H) = 1.2*U*_eq_(N).

### Crystal data

**Table:**

C_15_H_16_N_2_S_3_	*F*(000) = 672
*M**_r_* = 320.48	*D*_x_ = 1.377 Mg m^−^^3^
Monoclinic, *P*2_1_/*c*	Cu *K*α radiation, λ = 1.54182 Å
*a* = 5.6956 (4) Å	Cell parameters from 3915 reflections
*b* = 14.3424 (9) Å	θ = 3.1–71.3°
*c* = 18.9255 (11) Å	µ = 4.30 mm^−^^1^
β = 90.263 (5)°	*T* = 150 K
*V* = 1545.98 (17) Å^3^	Prism, yellow
*Z* = 4	0.15 × 0.10 × 0.06 mm

### Data collection

**Table:**

Oxford Diffraction Xcaliber Eos Gemini diffractometer	2830 independent reflections
Radiation source: fine-focus sealed tube	2506 reflections with *I* > 2σ(*I*)
Graphite monochromator	*R*_int_ = 0.023
Detector resolution: 16.1952 pixels mm^-1^	θ_max_ = 71.3°, θ_min_ = 3.9°
ω scans	*h* = −6→6
Absorption correction: multi-scan (*CrysAlis PRO*; Agilent, 2011)	*k* = −17→17
*T*_min_ = 0.774, *T*_max_ = 1.000	*l* = −16→22
8463 measured reflections	

### Refinement

**Table:**

Refinement on *F*^2^	1 restraint
Least-squares matrix: full	Hydrogen site location: mixed
*R*[*F*^2^ > 2σ(*F*^2^)] = 0.039	H atoms treated by a mixture of independent and constrained refinement
*wR*(*F*^2^) = 0.109	*w* = 1/[σ^2^(*F*_o_^2^) + (0.0667*P*)^2^ + 0.9619*P*] where *P* = (*F*_o_^2^ + 2*F*_c_^2^)/3
*S* = 1.06	(Δ/σ)_max_ = 0.001
2830 reflections	Δρ_max_ = 0.49 e Å^−^^3^
186 parameters	Δρ_min_ = −0.33 e Å^−^^3^

### Special details

**Table:**

Geometry. All e.s.d.'s (except the e.s.d. in the dihedral angle between two l.s. planes) are estimated using the full covariance matrix. The cell e.s.d.'s are taken into account individually in the estimation of e.s.d.'s in distances, angles and torsion angles; correlations between e.s.d.'s in cell parameters are only used when they are defined by crystal symmetry. An approximate (isotropic) treatment of cell e.s.d.'s is used for estimating e.s.d.'s involving l.s. planes.

### Fractional atomic coordinates and isotropic or equivalent isotropic displacement parameters (Å^2^)

**Table:**

	*x*	*y*	*z*	*U*_iso_*/*U*_eq_	
S1	0.51174 (9)	0.72659 (3)	0.42082 (3)	0.01620 (16)	
S2	0.71326 (10)	0.92093 (3)	0.43627 (3)	0.02009 (17)	
S3	−0.06660 (10)	0.60844 (4)	0.53247 (3)	0.02183 (17)	
N1	0.3513 (3)	0.85288 (12)	0.50603 (10)	0.0179 (4)	
H1N	0.339 (5)	0.9097 (9)	0.5222 (13)	0.021*	
N2	0.1971 (3)	0.78079 (12)	0.51907 (10)	0.0170 (4)	
C1	0.5185 (4)	0.83886 (14)	0.45784 (12)	0.0171 (4)	
C2	0.0454 (4)	0.79016 (15)	0.56846 (12)	0.0179 (5)	
C2'	0.0181 (5)	0.87383 (16)	0.61556 (13)	0.0258 (5)	
H2'1	0.1735	0.8977	0.6287	0.039*	
H2'2	−0.0675	0.8559	0.6583	0.039*	
H2'3	−0.0696	0.9224	0.5904	0.039*	
C3	−0.1146 (4)	0.71133 (15)	0.57733 (12)	0.0174 (4)	
C4	−0.3185 (4)	0.56050 (16)	0.56418 (13)	0.0237 (5)	
H4	−0.3703	0.4993	0.5530	0.028*	
C5	−0.4368 (4)	0.61936 (17)	0.60721 (13)	0.0243 (5)	
H5	−0.5818	0.6033	0.6286	0.029*	
C6	−0.3258 (4)	0.70718 (14)	0.61767 (12)	0.0175 (5)	
H6	−0.3834	0.7557	0.6470	0.021*	
C7	0.7561 (4)	0.73279 (15)	0.35948 (12)	0.0184 (5)	
H7A	0.7341	0.7856	0.3265	0.022*	
H7B	0.9052	0.7421	0.3856	0.022*	
C8	0.7625 (4)	0.64185 (14)	0.31920 (11)	0.0165 (4)	
C9	0.5788 (4)	0.61510 (15)	0.27450 (12)	0.0186 (5)	
H9	0.4473	0.6551	0.2684	0.022*	
C10	0.5867 (4)	0.53072 (15)	0.23898 (12)	0.0188 (5)	
H10	0.4602	0.5136	0.2087	0.023*	
C11	0.7773 (4)	0.47032 (15)	0.24688 (11)	0.0179 (5)	
C11'	0.7835 (4)	0.37808 (16)	0.20945 (14)	0.0259 (5)	
H11A	0.8516	0.3863	0.1624	0.039*	
H11B	0.6235	0.3537	0.2048	0.039*	
H11C	0.8795	0.3341	0.2367	0.039*	
C12	0.9610 (4)	0.49777 (15)	0.29108 (12)	0.0179 (5)	
H12	1.0934	0.4581	0.2968	0.022*	
C13	0.9533 (4)	0.58213 (15)	0.32682 (12)	0.0177 (5)	
H13	1.0800	0.5993	0.3569	0.021*	

### Atomic displacement parameters (Å^2^)

**Table:**

	*U*^11^	*U*^22^	*U*^33^	*U*^12^	*U*^13^	*U*^23^
S1	0.0185 (3)	0.0122 (3)	0.0180 (3)	−0.00068 (18)	0.0030 (2)	−0.00096 (18)
S2	0.0217 (3)	0.0135 (3)	0.0251 (3)	−0.0034 (2)	0.0057 (2)	−0.0019 (2)
S3	0.0233 (3)	0.0177 (3)	0.0244 (3)	−0.0018 (2)	−0.0013 (2)	−0.0007 (2)
N1	0.0208 (10)	0.0120 (8)	0.0209 (10)	−0.0019 (7)	0.0041 (8)	−0.0017 (7)
N2	0.0180 (9)	0.0139 (8)	0.0192 (9)	−0.0007 (7)	0.0008 (7)	0.0009 (7)
C1	0.0205 (11)	0.0131 (10)	0.0176 (11)	0.0012 (8)	−0.0015 (9)	−0.0002 (8)
C2	0.0188 (11)	0.0159 (10)	0.0190 (11)	0.0000 (8)	−0.0014 (9)	−0.0011 (8)
C2'	0.0298 (13)	0.0226 (11)	0.0251 (13)	−0.0066 (10)	0.0095 (10)	−0.0053 (10)
C3	0.0186 (11)	0.0169 (10)	0.0168 (11)	−0.0001 (8)	−0.0023 (9)	−0.0008 (8)
C4	0.0232 (12)	0.0205 (11)	0.0272 (13)	−0.0058 (9)	−0.0092 (10)	0.0064 (9)
C5	0.0185 (12)	0.0320 (13)	0.0225 (12)	−0.0039 (10)	−0.0024 (9)	0.0099 (10)
C6	0.0182 (11)	0.0146 (10)	0.0198 (11)	0.0005 (8)	−0.0076 (9)	0.0023 (8)
C7	0.0181 (11)	0.0168 (10)	0.0204 (11)	−0.0022 (8)	0.0061 (9)	−0.0006 (8)
C8	0.0178 (11)	0.0160 (10)	0.0157 (10)	−0.0023 (8)	0.0052 (8)	0.0008 (8)
C9	0.0169 (11)	0.0199 (10)	0.0190 (11)	0.0026 (8)	0.0012 (9)	0.0026 (8)
C10	0.0165 (11)	0.0230 (11)	0.0167 (11)	−0.0019 (9)	−0.0008 (9)	0.0002 (9)
C11	0.0194 (11)	0.0179 (10)	0.0164 (11)	−0.0014 (8)	0.0038 (9)	−0.0009 (8)
C11'	0.0259 (13)	0.0222 (11)	0.0296 (13)	0.0003 (9)	0.0008 (10)	−0.0073 (10)
C12	0.0161 (11)	0.0179 (10)	0.0198 (11)	0.0026 (8)	0.0003 (9)	0.0011 (8)
C13	0.0154 (11)	0.0203 (10)	0.0175 (11)	−0.0016 (8)	−0.0008 (9)	0.0003 (8)

### Geometric parameters (Å, º)

**Table:**

S1—C1	1.756 (2)	C6—H6	0.9500
S1—C7	1.818 (2)	C7—C8	1.511 (3)
S2—C1	1.670 (2)	C7—H7A	0.9900
S3—C4	1.703 (2)	C7—H7B	0.9900
S3—C3	1.725 (2)	C8—C13	1.391 (3)
N1—C1	1.337 (3)	C8—C9	1.396 (3)
N1—N2	1.379 (3)	C9—C10	1.385 (3)
N1—H1N	0.874 (10)	C9—H9	0.9500
N2—C2	1.283 (3)	C10—C11	1.396 (3)
C2—C3	1.462 (3)	C10—H10	0.9500
C2—C2'	1.503 (3)	C11—C12	1.393 (3)
C2'—H2'1	0.9800	C11—C11'	1.501 (3)
C2'—H2'2	0.9800	C11'—H11A	0.9800
C2'—H2'3	0.9800	C11'—H11B	0.9800
C3—C6	1.429 (3)	C11'—H11C	0.9800
C4—C5	1.354 (4)	C12—C13	1.387 (3)
C4—H4	0.9500	C12—H12	0.9500
C5—C6	1.423 (3)	C13—H13	0.9500
C5—H5	0.9500		
			
C1—S1—C7	101.21 (10)	C8—C7—S1	107.47 (14)
C4—S3—C3	92.08 (12)	C8—C7—H7A	110.2
C1—N1—N2	117.73 (17)	S1—C7—H7A	110.2
C1—N1—H1N	115.8 (18)	C8—C7—H7B	110.2
N2—N1—H1N	125.9 (18)	S1—C7—H7B	110.2
C2—N2—N1	118.90 (18)	H7A—C7—H7B	108.5
N1—C1—S2	122.48 (16)	C13—C8—C9	118.5 (2)
N1—C1—S1	113.31 (16)	C13—C8—C7	120.0 (2)
S2—C1—S1	124.21 (14)	C9—C8—C7	121.5 (2)
N2—C2—C3	115.14 (19)	C10—C9—C8	120.5 (2)
N2—C2—C2'	126.0 (2)	C10—C9—H9	119.7
C3—C2—C2'	118.9 (2)	C8—C9—H9	119.7
C2—C2'—H2'1	109.5	C9—C10—C11	121.1 (2)
C2—C2'—H2'2	109.5	C9—C10—H10	119.4
H2'1—C2'—H2'2	109.5	C11—C10—H10	119.4
C2—C2'—H2'3	109.5	C12—C11—C10	118.0 (2)
H2'1—C2'—H2'3	109.5	C12—C11—C11'	120.9 (2)
H2'2—C2'—H2'3	109.5	C10—C11—C11'	121.1 (2)
C6—C3—C2	128.3 (2)	C11—C11'—H11A	109.5
C6—C3—S3	111.28 (16)	C11—C11'—H11B	109.5
C2—C3—S3	120.31 (17)	H11A—C11'—H11B	109.5
C5—C4—S3	112.48 (18)	C11—C11'—H11C	109.5
C5—C4—H4	123.8	H11A—C11'—H11C	109.5
S3—C4—H4	123.8	H11B—C11'—H11C	109.5
C4—C5—C6	114.4 (2)	C13—C12—C11	120.9 (2)
C4—C5—H5	122.8	C13—C12—H12	119.5
C6—C5—H5	122.8	C11—C12—H12	119.5
C5—C6—C3	109.7 (2)	C12—C13—C8	120.9 (2)
C5—C6—H6	125.1	C12—C13—H13	119.6
C3—C6—H6	125.1	C8—C13—H13	119.6
			
C1—N1—N2—C2	175.2 (2)	C2—C3—C6—C5	175.4 (2)
N2—N1—C1—S2	179.03 (15)	S3—C3—C6—C5	−1.4 (2)
N2—N1—C1—S1	−1.3 (3)	C1—S1—C7—C8	−176.13 (15)
C7—S1—C1—N1	179.94 (17)	S1—C7—C8—C13	−115.3 (2)
C7—S1—C1—S2	−0.39 (17)	S1—C7—C8—C9	63.9 (2)
N1—N2—C2—C3	178.49 (18)	C13—C8—C9—C10	0.3 (3)
N1—N2—C2—C2'	−0.5 (3)	C7—C8—C9—C10	−178.9 (2)
N2—C2—C3—C6	−167.3 (2)	C8—C9—C10—C11	0.1 (3)
C2'—C2—C3—C6	11.7 (3)	C9—C10—C11—C12	−0.6 (3)
N2—C2—C3—S3	9.2 (3)	C9—C10—C11—C11'	178.8 (2)
C2'—C2—C3—S3	−171.73 (17)	C10—C11—C12—C13	0.7 (3)
C4—S3—C3—C6	0.80 (17)	C11'—C11—C12—C13	−178.7 (2)
C4—S3—C3—C2	−176.29 (18)	C11—C12—C13—C8	−0.3 (3)
C3—S3—C4—C5	0.04 (19)	C9—C8—C13—C12	−0.2 (3)
S3—C4—C5—C6	−0.9 (3)	C7—C8—C13—C12	179.1 (2)
C4—C5—C6—C3	1.5 (3)		

### Hydrogen-bond geometry (Å, º)

Cg1 and Cg2 are the centroids of the S3,C3–C6 and C8–C13 rings, respectively.

**Table:**

*D*—H···*A*	*D*—H	H···*A*	*D*···*A*	*D*—H···*A*
N1—H1*N*···S2^i^	0.87 (2)	2.57 (2)	3.4433 (18)	176 (3)
C2′—H2′2···*Cg*2^ii^	0.98	2.85	3.616 (3)	138
C12—H12···*Cg*1^iii^	0.95	2.89	3.560 (2)	130

Symmetry codes: (i) −*x*+1, −*y*+2, −*z*+1; (ii) *x*−1, −*y*+1/2, *z*−1/2; (iii) −*x*+1, −*y*+1, −*z*+1.

## References

[bb1] Agilent (2011). *CrysAlis PRO*. Agilent Technologies, Yarnton, England.

[bb2] Brandenburg, K. (2006). *DIAMOND*. Crystal Impact GbR, Bonn, Germany.

[bb3] Chan, M.-H. E., Crouse, K. A., Tarafder, M. T. H. & Yamin, B. M. (2003). *Acta Cryst.* E**59**, o628–o629.

[bb4] Farrugia, L. J. (2012). *J. Appl. Cryst.* **45**, 849–854.

[bb5] Sheldrick, G. M. (2015). *Acta Cryst.* C**71**, 3–8.

[bb6] Tarafder, M. T. H., Khoo, T.-J., Crouse, K. A., Ali, A. M., Yamin, B. M. & Fun, H.-K. (2002). *Polyhedron*, **21**, 2691–2698.

[bb7] Westrip, S. P. (2010). *J. Appl. Cryst.* **43**, 920–925.

