# Supplementary figures and images for: Crystal structure of 4-methyl­benzyl N′-[(thio­phen-2-yl)methyl­idene]hydrazinecarbodi­thio­ate
Source: Acta Crystallogr E Crystallogr Commun. 2015 Jun 13;71(Pt 7):o475–6. doi: 10.1107/S205698901501107X (PMC4518925; doi:10.1107/S205698901501107X)

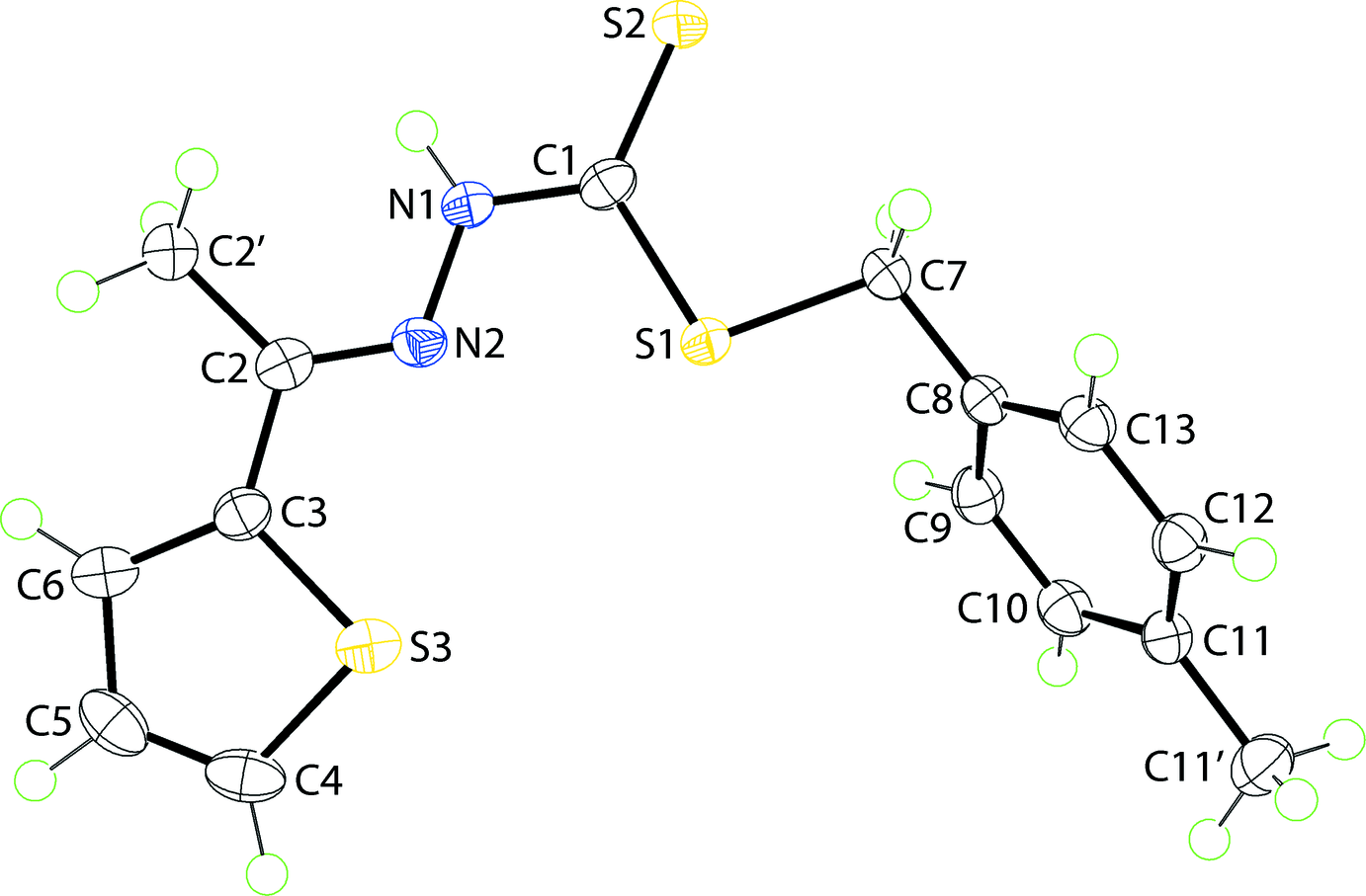

Supplement: Supplementary file 3 [file e-71-0o475-fig1.tif]

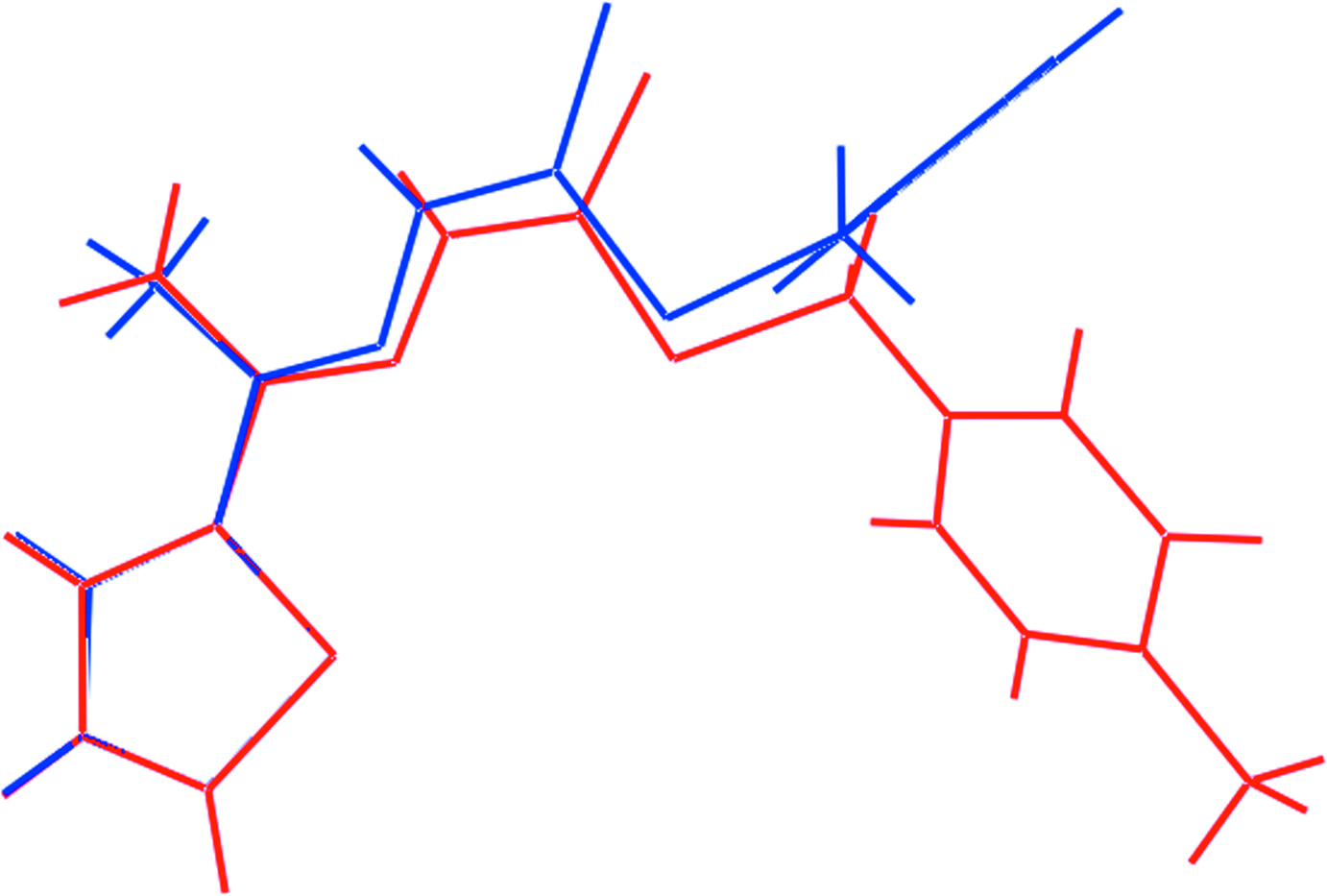

Supplement: Supplementary file 4 [file e-71-0o475-fig2.tif]

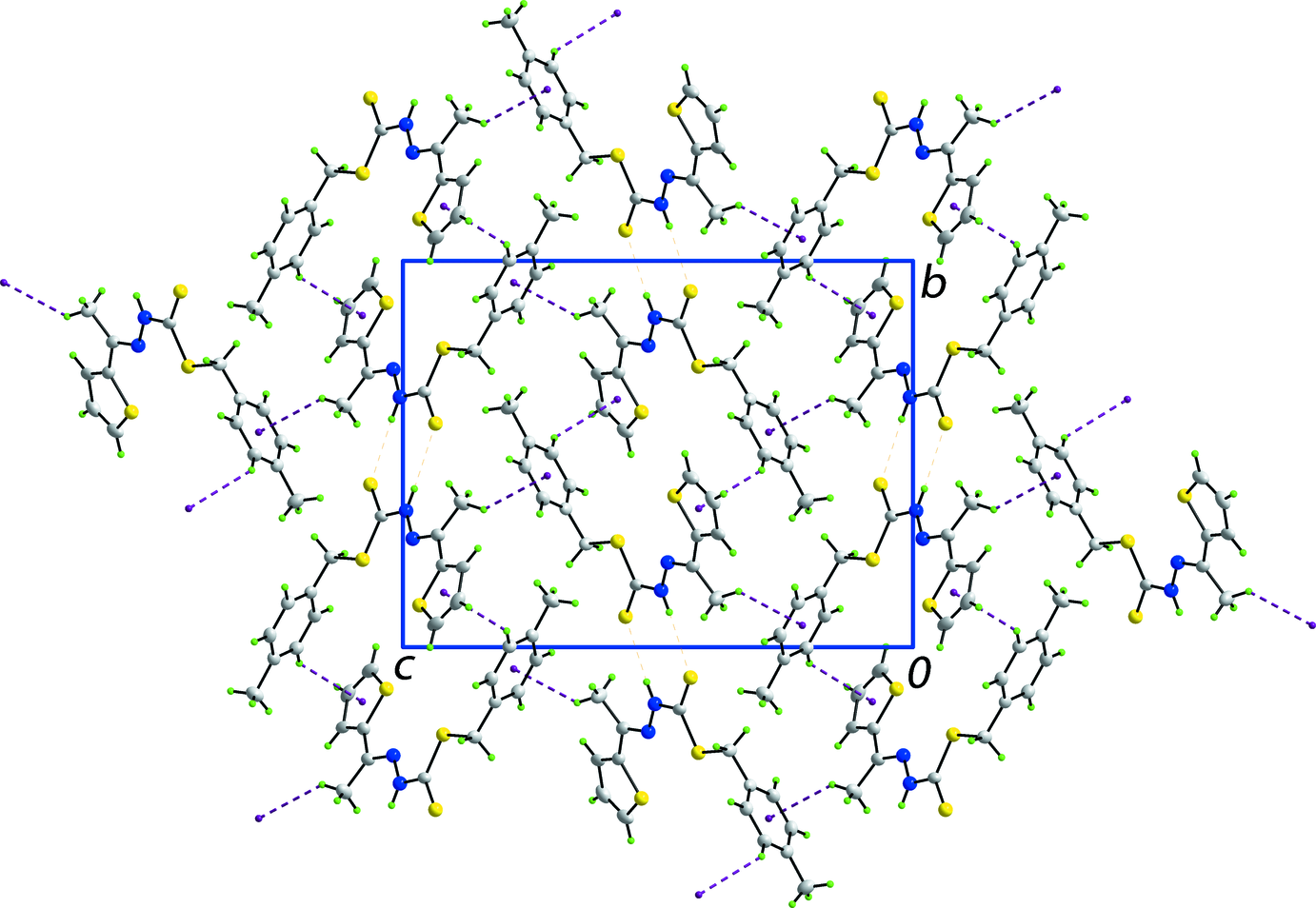

Supplement: Supplementary file 5 [file e-71-0o475-fig3.tif]
